# Impact of Tumor Site and Adjuvant Radiotherapy on Survival of Patients with Adenoid Cystic Carcinoma: A SEER Database Analysis

**DOI:** 10.3390/cancers13040589

**Published:** 2021-02-03

**Authors:** Jason Tasoulas, Kimon Divaris, Stamatios Theocharis, Douglas Farquhar, Colette Shen, Trevor Hackman, Antonio L. Amelio

**Affiliations:** 1 First Department of Pathology, Medical School, National and Kapodistrian University of Athens, 11527 Athens, Greece; tasoulas@email.unc.edu (J.T.); stamtheo@med.uoa.gr (S.T.); 2 Lineberger Comprehensive Cancer Center, School of Medicine, University of North Carolina at Chapel Hill, Chapel Hill, NC 27599, USA; 3 Division of Oral and Craniofacial Health Sciences, Adams School of Dentistry, University of North Carolina at Chapel Hill, Chapel Hill, NC 27599-7455, USA; 4 Department of Epidemiology, Gillings School of Global Public Health, University of North Carolina at Chapel Hill, Chapel Hill, NC 27599, USA; Kimon_Divaris@unc.edu; 5 Division of Pediatric and Public Health, Adams School of Dentistry, University of North Carolina at Chapel Hill, Chapel Hill, NC 27599-7455, USA; 6 Department of Otolaryngology/Head and Neck Surgery, School of Medicine, University of North Carolina at Chapel Hill, Chapel Hill, NC 27599, USA; Douglas.Farquhar@unchealth.unc.edu (D.F.); trevor_hackman@med.unc.edu (T.H.); 7 Department of Radiation Oncology, University of North Carolina at Chapel Hill, Chapel Hill, NC 27599, USA; colette_shen@med.unc.edu; 8 Department of Cell Biology and Physiology, School of Medicine, University of North Carolina at Chapel Hill, Chapel Hill, NC 27599, USA; 9 Cancer Cell Biology Program, Lineberger Comprehensive Cancer Center, School of Medicine, University of North Carolina at Chapel Hill, Chapel Hill, NC 27599, USA

**Keywords:** adenoid cystic carcinoma, adjuvant radiotherapy, submandibular salivary gland, prognosis, surveillance, epidemiology, and end results (SEER) database

## Abstract

**Simple Summary:**

Adenoid cystic carcinoma (ACC) is a rare but lethal salivary gland tumor, comprising less than 1% of head and neck neoplasms. It can affect several sites in the head and neck region, but the potential role of tumor site on survival and treatment response remains unclear and, to this day, the role that tumor location plays in treatment planning and survival has not been considered. We sought to address these knowledge gaps via analyses of the Surveillance, Epidemiology, and End Results (SEER) database. Our analysis identified that patients with submandibular gland adenoid cystic carcinomas, particularly those with late-stage disease, had worse survival compared to other sites and benefited the most from adjuvant radiotherapy. In contrast, early-stage and non-submandibular adenoid cystic carcinoma patients showed no survival benefit from adjuvant radiotherapy (aRT). These results underscore the role of tumor site on treatment response and survival of patients with head and neck ACC.

**Abstract:**

Adenoid cystic carcinoma (ACC) is a rare salivary gland tumor, displaying aggressive behavior with frequent recurrence and metastasis. Little information exists regarding the impact of clinicopathological parameters and adjuvant radiotherapy (aRT) on ACC disease specific (DSS) and overall survival (OS). We extracted demographic, treatment, and survival information of 1439 patients with major or minor intraoral salivary gland ACC from the Surveillance, Epidemiology, and End Results (SEER) database. The associations between tumor characteristics and aRT with OS and DSS were estimated using hazard ratios (HR) and 95% confidence intervals (CI). Submandibular gland ACCs had the worst prognosis (adjusted DSS HR = 1.48; 95% CI = 0.99–2.20, compared to parotid), and this difference was more pronounced among patients with advanced-stage tumors (adjusted DSS HR = 1.93; 95% CI = 1.13–3.30). aRT was associated with increased overall survival only among stage III submandibular ACC patients (HR = 0.64; 95% CI = 0.42–0.98) and had no benefit in any other group. In conclusion, submandibular gland ACC carries a worse prognosis than other gland subsites and may benefit from aRT. The different outcomes between submandibular gland and other major or minor gland ACCs warrant further mechanistic investigation.

## 1. Introduction

Adenoid cystic carcinoma (ACC) is a rare malignancy of the salivary glands [1], comprising less than 1% of the head and neck neoplasms. Less frequently, ACCs have been reported in the trachea or the lacrimal glands, among other sites of the sinonasal and respiratory tract [2,3]. According to the Surveillance, Epidemiology, and End Results (SEER) database, ACC incidence in the US has declined during the last decades [4]. However, significant geographic variation exists; ACC has been reported as the most common salivary gland malignancy in western Europe, whereas in the US it is ranked 3rd after mucoepidermoid carcinoma and polymorphous adenocarcinoma [3].

Clinically, ACC tumors present as a slow-growing mass or mucosal ulceration. The tumor is unpredictable and characterized by local and perineural invasion, indolent growth, local relapse, and over time (usually a decade or more) distant metastases. Given the preponderance of perineural invasion and advanced T classification at presentation, surgical resection followed by radiation is the standard of care for the management of the majority ACC cases regardless of anatomic location or gland subtype. However, the prognostic impact of this treatment paradigm remains poorly understood.

Little is known regarding specific risk factors, but significant progress has been made in terms of unraveling ACC pathobiology [5]. Recent evidence supports an etiologic link between the MYB-NFIB, MYBL1-NFIB, or MYBL1-RAD51 gene fusions and ACC [5,6,7]. However, the impact of ACC clinicopathological parameters (anatomic tumor site, major gland subtype, sex, race, age) on survival is relatively understudied. Some evidence suggests that intraoral minor gland ACC (m-ACC) has a better prognosis than major gland (M-ACC) and ACC of the sinonasal tract [4,8]. Other reports suggest that these anatomic location differences of ACC tumors do not affect their prognosis [9,10]. Additionally, the role of adjuvant radiotherapy (aRT) in ACC management, while discussed and debated for decades, remains controversial [4,9,10,11,12,13,14,15,16,17]. For example, aRT is recommended for the management of late-stage disease (stage III or IV), but recent evidence suggests that it may also be associated with improved survival in early-stage disease [10]. While aRT has been included in the latest National Comprehensive Cancer Network (NCCN) guidelines, it remains unclear whether aRT can improve survival and under which clinicopathological parameters its implementation offers maximum clinical benefit [18].

We sought to address these knowledge gaps regarding the impact of clinicopathological parameters and aRT on ACC survival, overall, as well as for early- and late-stage disease, via an analysis of the Surveillance, Epidemiology, and End Results (SEER) database.

## 2. Results

**Figure 1:**
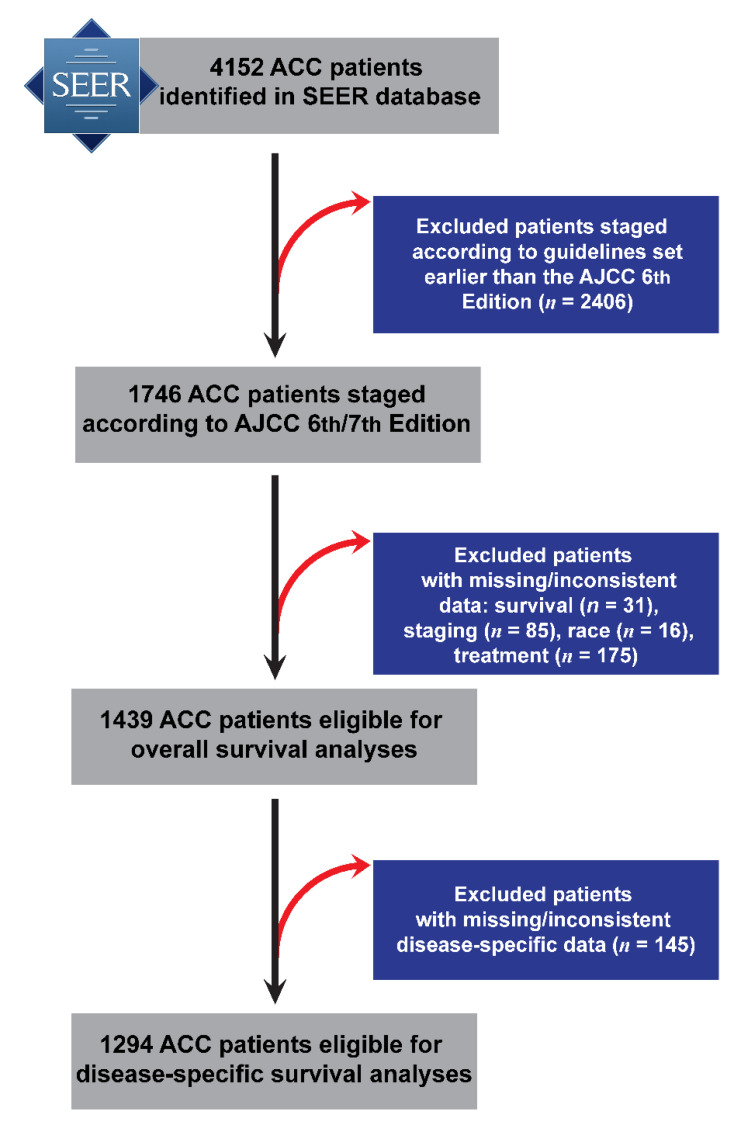
Flowchart of major and minor adenoid cystic carcinoma (ACC) patient selection and exclusions from the Surveillance, Epidemiology, and End Results (SEER) database (2004–2015) resulting to the analytical sample that was carried forward to disease-specific survival analysis. AJCC: American Joint Committee on Cancer.

Initially, 4152 ACC cases were retrieved from the SEER database, including 1653 with m-ACC and with 2499 M-ACC. Demographic (age, sex, race), clinicopathological (TNM stage), and overall survival (OS) information was available for 1439 individuals diagnosed between 2004 and 2015 (out of which 1294 had disease specific survival (DSS) information), and this sample was carried forward to survival analyses (Figure 1). The sample was predominantly female (59%) with most cases being early-stage (I and II) (58%) (Table 1). M-ACCs were more common among younger and female patients. Significant differences were also found in TNM stage presentation between M-ACC and m-ACC, as well as between M-ACC subtypes (parotid, submandibular, sublingual) (Table 2). Adjuvant radiotherapy was common such that nearly three-quarters of patients received aRT, with younger and late-stage patients significantly more likely to have received it compared to older and early-stage disease patients (*p* < 0.0005) (Table 3).

### 2.1. Submandibular Gland Has the Worst Prognosis among Advanced-Stage Adenoid Cystic Carcinoma Tumors

**Figure 2:**
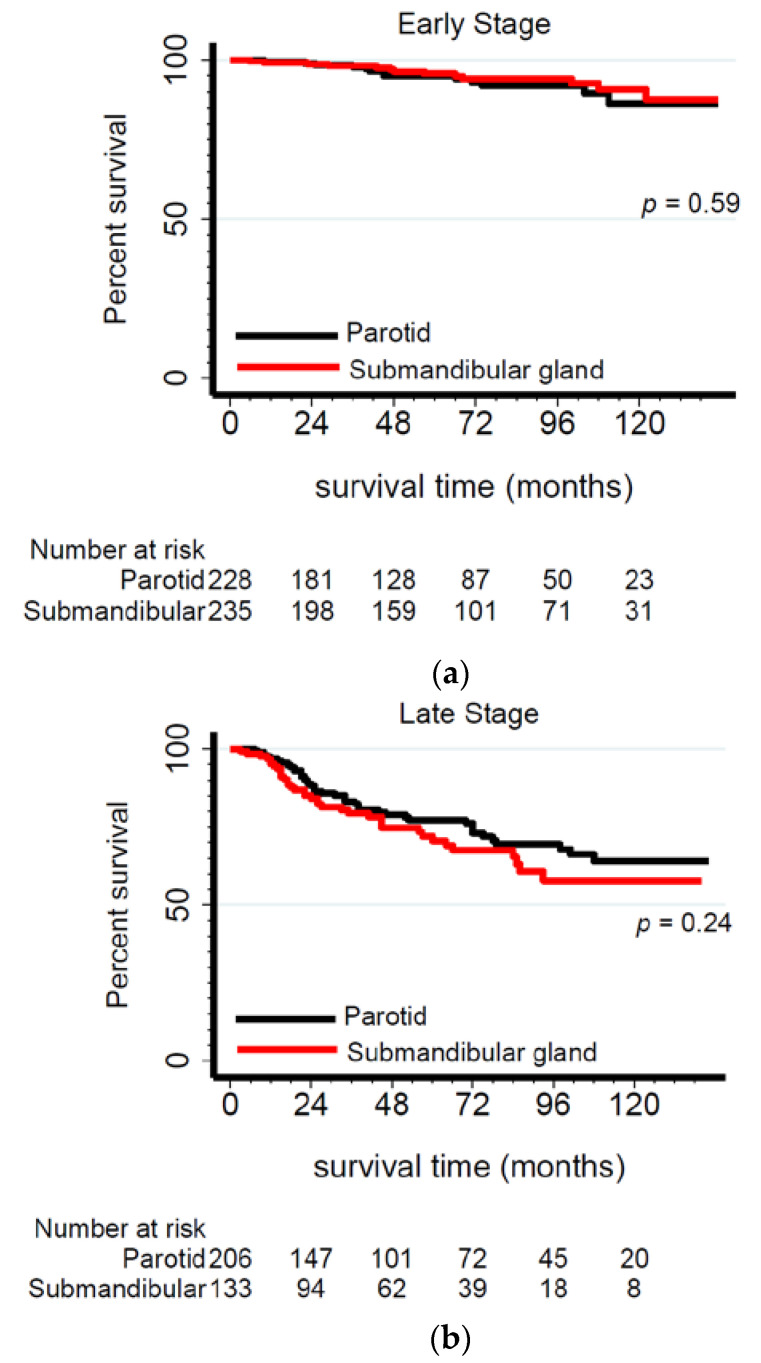
Kaplan–Meier survival curves for early-stage (stage I&II) (**a**) versus late-stage (stage III&IV) (**b**) ACC patients according to major gland type. Note: Sublingual gland ACC patients were not included in this analysis due to the small number of cases.

**Figure 3:**
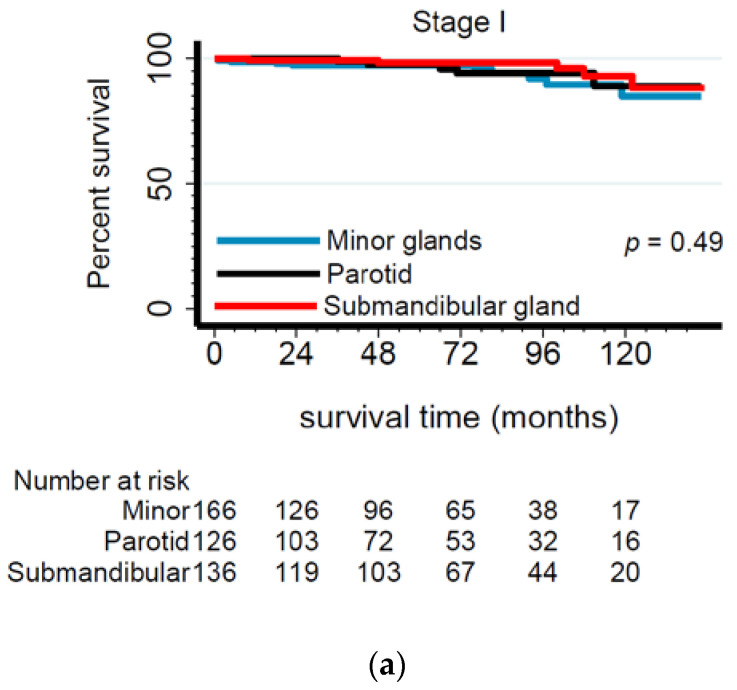

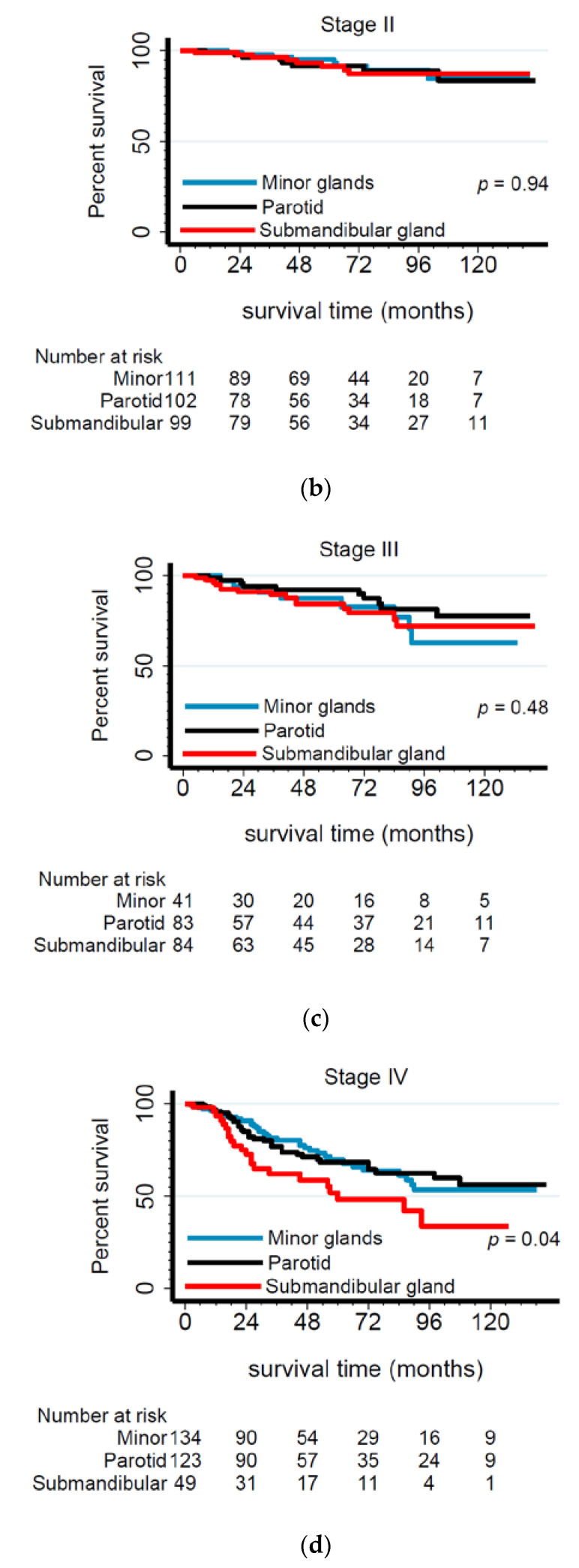
Kaplan–Meier survival curves for stage I (**a**), II (**b)**, III (**c**), IV (**d**) ACC patients according to gland type. Note: Sublingual gland ACC patients were not included in this analysis due to the small number of cases.

There were minimal overall differences in 5-year survival rates obtained from Kaplan–Meier analysis according to anatomic site and gland type, i.e., OS—parotid: 81%, submandibular: 82%, sublingual: 82%, and minor: 81%; DSS—parotid: 86%, submandibular: 87%, sublingual: 90%, and minor: 88%. However, when considering late-stage tumors only, submandibular ACC had significantly lower 5-year survival (OS: 65%, DSS: 71%) than other tumors (e.g., parotid, OS: 73%, DSS: 77%) (Figure 2 and Figure 3). When accounting for demographics, tumor and treatment characteristics in Cox regression modeling showed that submandibular gland ACCs remained the most lethal among late-stage tumors (Table 4) and that this difference was more pronounced among patients with stage IV tumors. Specifically, this difference was almost double that of submandibular gland ACCs, exhibiting 93% (DSS) higher hazard ratios (Table 5).

### 2.2. Adjuvant Radiotherapy Confers Survival Advantage Only among Late-Stage Submandibular Tumors

**Figure 4:**
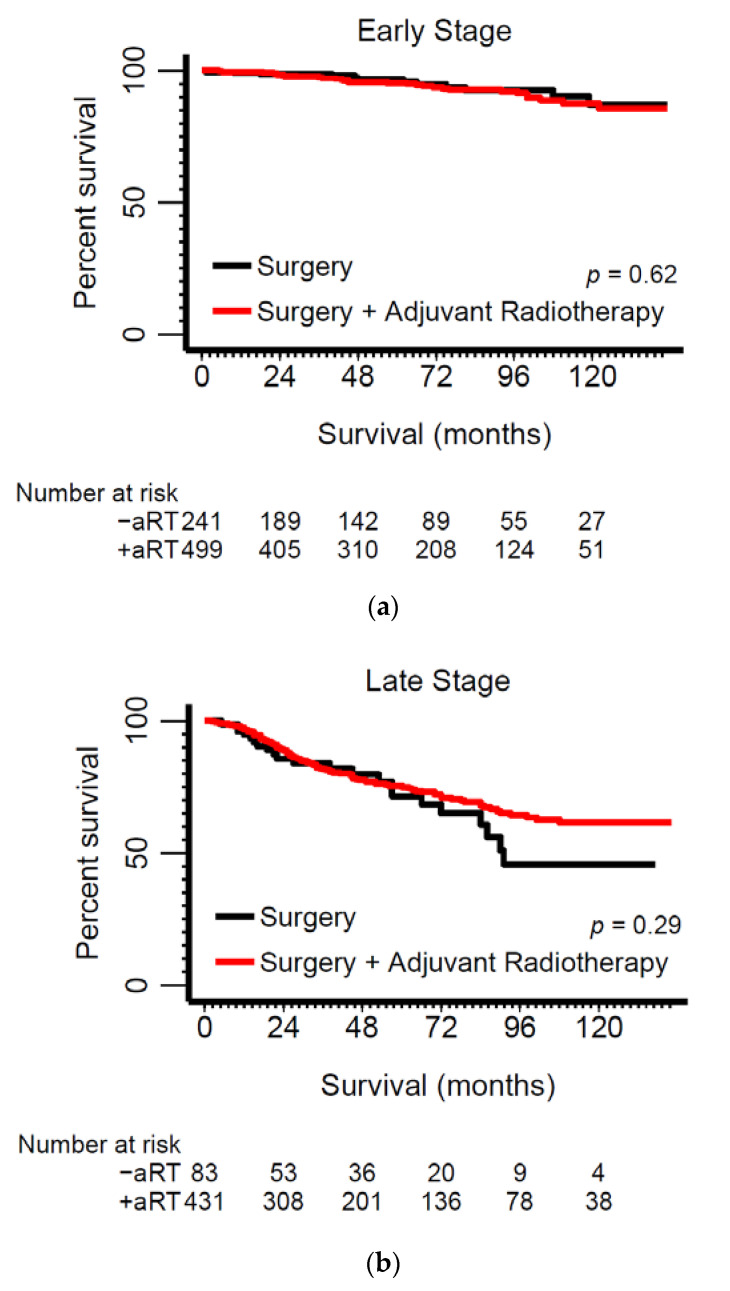
Kaplan–Meier survival curves for early-stage (stage I & II) (**a**) versus late-stage (stage III & IV) (**b**) ACC patients according to according to treatment via surgery versus surgery and adjuvant radiotherapy (aRT).

Most early-stage patients (67%) received aRT; however, there was no aRT-associated survival benefit in this group (Table 4; Figure 4a). Among late-stage patients, aRT administration was associated with 38% and 16% lower hazard ratios for overall and disease-specific death, respectively (Table 4; Figure 4b). Tumor site, gland type, and stage-stratified analyses (Table 6) revealed survival benefits associated with aRT among patients with stage ΙΙΙ submandibular tumors (Stage III OS: adjusted hazard ratio (HR) = 0.35; 95% CI = 0.15–0.85). Although HRs were not estimable or were imprecise in some strata with small numbers of cases (e.g., sublingual tumors), we found that except for late-stage submandibular tumors there was no evidence of aRT-associated survival benefit associated with any other major or minor salivary gland ACC.

## 3. Discussion

In this report, we present the results of a comprehensive survival analysis of m- and M-ACC patients in SEER, wherein we sought to determine the impact of clinicopathological tumor parameters and receipt of aRT on survival. Among patients with advanced-stage disease, submandibular ACC had significantly worse prognosis than minor and parotid salivary gland tumors. Moreover, we found that aRT is not associated with survival benefits in early-stage or m-ACC, whereas it does confer increased survival in late-stage submandibular cases. These results add to the evolving knowledge base of head and neck ACC-related outcomes and suggest the existence of heterogeneity in survival and response to aRT according to tumor site and stage. The observational associations reported here provide motivation and set the stage for future mechanistic studies investigating the biologic basis underlying these observed differences in salivary gland (SG) ACC prognosis and response to treatment.

It is noteworthy that we only detected differences in survival between minor and major salivary gland ACC after stratifying M-ACC according to gland subtype. Moreover, this difference was evident only among late-stage disease and statistically significant among stage IV. This indicates the existence of substantial heterogeneity within M-ACC tumors—indeed, submandibular ACC had considerably worse prognosis in terms of OS and DSS compared to parotid and sublingual sites, after accounting for all important covariates (i.e., age, sex, race, TNM stage and receipt of aRT). The existing evidence on this topic is mixed: nasopharyngeal and M-ACCs have been reported as having worse prognosis compared to intraoral m-ACCs [4,8] and better prognosis than intraoral m-ACCs [19] or no difference [10,20,21]. Previous reports demonstrating that major salivary gland ACCs, specifically submandibular tumors, metastasize more often to distant organs [14,22,23,24]. Moreover, a recent study comparing orthotopic submandibular (PDOX) and subcutaneous (PDX) patient-derived ACC xenografts reported significantly higher tumor growth rates and, more importantly, retention of histopathologic features with the PDOX model [25]. These findings support the notion that anatomic tumor site influences a unique biological context present in M-ACCs that may influence the pathological behavior of submandibular gland ACCs [14,22,23,24,25]. A plausible mechanism may involve the tumor microenvironment (TME) [22]; high vascular density and numerous tumor-associated vessels are identified within submandibular M-ACCs, which could indicate a potential pathway for dissemination to distant sites. Additionally, interleukin (IL)-6 expression is higher in submandibular M-ACCs compared to other sites. Given the prominent role of IL-6 in microenvironmental signaling and tumor progression [26], this finding further strengthens the hypothesis that unique TME features of the submandibular gland could affect its prognosis and biological behavior. However, vascular remodeling in the context of carcinogenesis is far more complex and thus further research is required to unravel its potential role in ACC biology. Notably, submandibular gland has also been associated with worse survival compared to other major gland sites (HR = 1.21, 95% CI = 1.00–1.46) in a retrospective National Cancer Database (NCDB) pooled analysis of 2210 patients with salivary gland cancers [27]

Primary tumor management includes surgical excision and adjuvant radiotherapy. Although aRT is an established part of the therapeutic protocols for ACC [18], its impact on survival has been under debate. While in the present cohort, two-thirds of early-stage patients received aRT, our data did not provide support for any survival benefit in this group. However, aRT was associated with better survival in a subgroup that included more aggressive ACC cases—submandibular stage III ACCs. This observation lends further support to our hypothesis that the biological context of a given salivary gland influences their pathological behaviors and may promote heterogeneity, as evidenced by variable treatment responses. Treatment with aRT has been reported as having a positive impact on survival or better local control in some reports [9,10,12,13,15,16,17], while few studies have reported little or no benefit [4,11,14]. Our results suggest that aRT administration in patients with early-stage ACC is not associated with improved survival outcomes. SEER does not include measures of quality of life and thus this domain cannot be evaluated; however, the harms of overtreatment are widely acknowledged and thus should not be overlooked in treatment planning [28].

Differences in the reported associations emanating from database studies [4,10,14,21] can be attributed to study design variations and the inherent limitations of these secondary data. Earlier reports from the SEER database used patient data up to 2004 [14], 2007 [4], or 2013 [21], while our study included patients up to 2015. Importantly, all 1439 cases in our analytical sample were diagnosed between 2004–2015. Lloyd et al. and Shen et al., included cases of cribriform carcinoma (International Classification of Diseases for Oncology, 3rd ed. (ICD-O3) = 8201) while Lee et al. retrieved only M-ACC data from the NCDB [10,14,21]. We submit that the higher specificity and homogeneity of included neoplasms investigated in our study, and the larger sample size compared to previous reports, are elements augmenting both the validity and the precision of our reported estimates of association.

Despite its merits, our study has some limitations, most of which are inherent to a secondary data analysis of this nature. Databases like SEER [29], NCDB [30], and EUROCARE [31] allow retrospective analyses of large numbers of cancer cases that can illuminate subtle differences and important clinicopathological associations in otherwise rare and hard to study tumors. The retrospective study design limits the investigators’ ability to collect and analyze information that may be relevant to the tumor under study; for instance, we were unable to utilize information from histology reports (e.g., histological type, margin status, and perineural invasion status) as well as behavioral risk factor data (e.g., alcohol consumption and smoking). These parameters have been identified as independent prognostic factors associated with worse survival [32,33]. Importantly, no large cancer database, including SEER, captures information on patients’ quality of life, which would be an important addition to our aRT analysis. An additional issue, common to SEER analyses, is related to possibly heterogeneous neoplasm groupings [34]. To address this potential limitation, in this study we included cases with only salivary gland ACC diagnoses (ICD-O-3 = 8200). The small number of cases in certain subgroups prohibited further analysis of site and stage strata (e.g., minor gland ACC stage III). It is also important to acknowledge that the stage IV submandibular gland ACC findings were based on a relatively small number of 49 cases. We acknowledge that our study is limited by the lack of information on the criteria used to select aRT candidates and the treatment parameters of aRT (including dose/fractionation, definitive versus palliative intent) in the SEER database. Certainly, radiotherapy is a rapidly growing field with advances in technology that has transformed adjuvant cancer care, primarily with respect to improvements in morbidity. Even within the period studied (2004–2015), significant advances in radiotherapy technology have been made; however, differences in aRT selection criteria, techniques, and equipment were likely to impact toxicity and quality of life more than survival outcomes during that time frame. Additionally, pathologic variables such as margin status and perineural invasion, which are not recorded in the SEER database and thus not accounted for in the analysis, may have influenced the decision-making regarding aRT administration—many patients who receive radiation have tumors with high-risk features.

## 4. Materials and Methods

Data were extracted from the SEER database (1973–2015), including additional treatment fields [29], using SEER Stat 8.3.5 [35]. SEER is a publicly available database and thus Institutional Review Board approval was not required. The research algorithm was run in a case listing session and included ACC as defined by the World Health Organization (WHO) 2005 salivary gland tumor classification [36], based on the International Classification of Diseases for Oncology, 3rd ed. (ICD-O-3) system (ACC = 8200) [37]. Data were extracted for minor and major ACC, separately. The m-ACC group included only intraoral locations (Appendix A) while M-ACC included parotid, submandibular, and sublingual gland ACCs (Appendix B).

For each group, the following data fields were retrieved and coded as follows: patient ID, age (coded in tertiles: <55; 55–69; >69 years), sex (male, female), race (white, black, other), year of diagnosis, anatomic site (major gland, minor gland), major gland subtype (parotid, submandibular, sublingual), TNM stage (I through IV), surgery, aRT, and disease specific survival (DSS) and overall survival (OS), both measured in months. For this study, we considered data on ACC cases that were staged using the American Joint Committee on Cancer (AJCC) 6th and 7th editions, effectively 2004 and onward. Cases between 2004 and 2009 were staged according to the AJCC 6th edition while those in 2010 and onwards were staged using the AJCC 7th. No major revisions were done in the 7th edition, and thus cases classified according to the AJCC 6th and 7th editions were pooled. Patients with distant metastases (Stage IVC) or patients that did not receive surgery were excluded from the analysis. Patients that received radiotherapy before surgery or with unknown sequence to surgery were also excluded. We estimated two 5-year survival rates, for OS and DSS.

We used summary statistics, univariate, and bivariate methods (e.g., Kaplan–Meier survival analyses and bivariate tests with a conventional *p* < 0.05 statistical significance criterion) to describe and present the distribution of key patient and tumor characteristics, including survival in the analytical sample—overall and stratified by tumor type. The associations of tumor site and aRT with OS and DSS were estimated using hazard ratios (HR) and corresponding 95% confidence intervals (CI) obtained via Cox regression models adjusted for radiation, TNM stage, anatomic site, primary site, sex, age, and race. Stratified models were also constructed to investigate variations of aRT’s survival benefit according to stage, anatomic site, and major gland subtype. Statistical analyses were done with Stata 16.1 (StataCorp LP, College Station, TX, USA), and figures were produced using Prism 8.0 (GraphPad Software, San Diego, CA, USA).

## 5. Conclusions

Our SEER database analysis addresses a knowledge gap in our understanding of ACC prognosis according to tumor characteristics and receipt of aRT. We found that advanced-stage submandibular ACC had significantly worse prognosis than all other tumor sites. Moreover, despite that aRT administration was very common among early-stage patients in our cohort, we found no evidence of aRT benefit in terms of survival. Contrary, receipt of aRT was associated with better survival only among late-stage submandibular gland tumors.

Collectively, our results indicate that tumor site is a parameter that should be evaluated in SG ACC treatment planning. The survival or other benefits of aRT in early-stage disease will need to be well justified; otherwise, these patients may be able to be spared from unnecessary radiation and morbidity. These findings from our retrospective SEER database analysis will require independent validation in other cohorts and populations. Additional mechanistic studies may help elucidate the underlying biological link between submandibular TME and ACC clinical behavior. The submandibular and minor SG tumors are logical candidates for such mechanistic follow-ups, given the high and low degrees of aggressiveness, respectively, that they demonstrated in our analyses.

## Figures and Tables

**Table 1:** Demographic and tumor characteristics in patients diagnosed with salivary gland ACC in the SEER database (2004–2015).

	Entire Sample, *n* (%)	Anatomic Tumor Site, *n* (%)		
		Minor Gland	Major Gland	X^2^
				*p*
total	1439 (100)	507 (100)	932 (100)	
**Age group (years)**				0.114
<55	615 (43)	199 (39)	416 (45)	
55–69	472 (33)	181 (36)	291 (31)	
>69	352 (24)	127 (25)	225 (24)	
**Sex**				0.013
male	587 (41)	229 (45)	358 (38)	
female	852 (59)	278 (55)	574 (62)	
**Race**				0.234
white	1130 (79)	408 (80)	722 (77)	
black	139 (10)	49 (10)	90 (10)	
other	170 (12)	50 (10)	120 (13)	
**Stage** ^1^				<0.0005
I	465 (32)	181 (36)	284 (30)	
II	369 (26)	127 (25)	242 (26)	
III	249 (17)	46 (9)	203 (22)	
IV	356 (25)	153 (30)	203 (22)	
IVA	286 (20)	119 (23)	167 (18)	
IVB	59 (4)	30 (6)	29 (3)	
IV NOS	11 (1)	4 (1)	7 (1)	
**T-classification** ^1^				<0.0005
T1	488 (34)	189 (37)	299 (32)	
T2	401 (28)	137 (27)	264 (28)	
T3	234 (16)	40 (8)	194 (21)	
T4	315 (22)	141 (28)	174 (19)	
Tx	1 (0)	0 (0)	1 (0)	
**N-classification** ^1^				0.268
N0	1260 (88)	456 (90)	804 (86)	
N1	98 (7)	28 (6)	70 (8)	
N2	77 (5)	22 (4)	55 (6)	
N3	2 (0)	0 (0)	2 (0)	
Nx	2 (0)	1 (0)	1 (0)	

^1^ Clinicopathological (TNM) stage information was based on the 6th and 7th edition of American Joint Committee on Cancer. NOS: Not otherwise specified.

**Table 2:** Demographic and tumor characteristics in patients diagnosed with major salivary gland ACC in the SEER database (2004–2015).

	Major Salivary Gland Type, *n* (%)			
	Parotid	Submandibular	Sublingual	X^2^
				*p*
total	476 (100)	413 (100)	43 (100)	
**Age group (years)**				0.153
<55	220 (46)	183 (44)	13 (30)	
55–69	153 (32)	123 (30)	15 (35)	
>69	103 (22)	107 (26)	15 (35)	
**Sex**				0.885
male	183 (38)	157 (38)	18 (42)	
female	293 (62)	256 (62)	25 (58)	
**Race**				0.299
white	376 (79)	314 (76)	32 (74)	
black	49 (10)	38 (9)	3 (7)	
other	51 (11)	61 (15)	8 (19)	
**Stage** ^1^				<0.0005
I	134 (28)	147 (36)	3 (7)	
II	113 (24)	115 (28)	14 (33)	
III	91 (19)	96 (23)	16 (37)	
IV	138 (30)	55 (13)	10 (23)	
IVA	117 (25)	42 (10)	8 (19)	
IVB	17 (4)	11 (3)	1 (2)	
IV NOS	4 (1)	2 (0)	1 (2)	
**T-classification** ^1^				<0.0005
T1	144 (30)	151 (37)	4 (9)	
T2	120 (25)	130 (31)	14 (33)	
T3	84 (18)	95 (23)	15 (35)	
T4	128 (27)	36 (9)	10 (23)	
Tx	0 (0)	1 (0)	0 (0)	
**N-classification** ^1^				0.865
N0	411 (86)	358 (87)	35 (81)	
N1	39 (8)	27 (7)	4 (9)	
N2	24 (5)	27 (7)	4 (9)	
N3	1 (0)	1 (0)	0 (0)	
Nx	1 (0)	0 (0)	0 (0)	

^1^ TNM stage information was based on the 6th and 7th Edition of AJCC. NOS: Not otherwise specified.

**Table 3:** Receipt of adjuvant radiotherapy in patients diagnosed with salivary gland ACC in the SEER database (2004–2015).

	Receipt of Adjuvant Radiotherapy, *n* (%)		
	No	Yes	X^2^
			*p*
total	385 (100)	1054 (100)	
**Anatomic site**			<0.0005
minor	172 (45)	335 (32)	
major	213 (55)	719 (68)	
**Major gland subtype**			0.622
parotid	115 (54)	361 (50)	
submandibular	89 (42)	324 (45)	
sublingual	9 (4)	34 (5)	
**Age group (years)**			0.029
<55	86 (40)	330 (46)	
55–69	61 (29)	230 (32)	
>69	66 (31)	159 (22)	
Sex			0.406
male	87 (41)	271 (38)	
female	126 (59)	448 (62)	
**Race**			0.199
white	157 (74)	565 (79)	
black	27 (13)	63 (9)	
other	29 (14)	91 (13)	
**Stage**			0.001
I	88 (41)	196 (27)	
II	54 (25)	188 (26)	
III	38 (18)	165 (23)	
IV	33 (15)	170 (24)	
IVA	29 (14)	138 (19)	
IVB	2 (1)	27 (4)	
IV NOS	2 (1)	5 (1)	

**Table 4:** Association of tumor site, type, and stage with overall and disease-specific survival estimated with Cox regression modeling ^1^ in early-stage (stage I & II) vs. late-stage (stage III & IV) patients diagnosed with salivary gland ACC in the SEER database (2004–2015).

	Early-Stage (I & II)				Late-Stage (III & IV)			
	Overall Survival		Disease-Specific Survival ^2^		Overall Survival		Disease-Specific Survival ^2^	
	HR (95% CI)	*p*	HR (95% CI)	*p*	HR (95% CI)	*p*	HR (95% CI)	*p*
**Age group (years)**								
<55	referent		referent		referent		referent	
55–69	1.82 (0.98–3.38)	0.057	1.14 (0.49–2.64)	0.757	1.64 (1.05–2.58)	0.031	1.28 (0.75–2.19)	0.369
>69	5.97 (3.35–10.67)	<0.0005	2.18 (0.85–5.61)	0.105	2.29 (1.47–3.56)	<0.0005	1.64 (0.96–2.78)	0.068
**Sex**								
male	referent		referent		referent		Referent	
female	0.77 (0.49–1.22)	0.269	0.56 (0.27–1.17)	0.126	1.21 (0.84–1.74)	0.302	1.43 (0.91–2.26)	0.125
**Race**								
white	Referent		referent		referent		referent	
black	1.15 (0.55–2.41)	0.713	1.22 (0.36–4.13)	0.745	0.86 (0.45–1.65)	0.658	0.73 (0.33–1.63)	0.449
other	0.84 (0.40–1.77)	0.642	1.58 (0.62–4.03)	0.334	1.20 (0.73–1.95)	0.476	1.60 (0.91–2.85)	0.105
**Adjuvant Radiotherapy**								
no	Referent		referent		referent		referent	
yes	1.06 (0.63–1.78)	0.835	1.49 (0.60–3.71)	0.390	0.62 (0.40–0.94)	0.026	0.75 (0.41–1.39)	0.368
**Anatomic site**								
minor	referent		referent		referent		referent	
major	0.92 (0.63–1.33)	0.645	0.88 (0.48–1.62)	0.673	0.81 (0.60–1.09)	0.155	0.84 (0.58–1.22)	0.367
**Major gland subtype**								
parotid	referent		referent		referent		referent	
submandibular	0.88 (0.55–1.41)	0.594	0.75 (0.34–1.63)	0.462	1.22 (0.85–1.74)	0.276	1.29 (0.83–2.01)	0.257
sublingual	2.00 (0.69–5.78)	0.199	3.12 (0.96–10.21)	0.059	0.59 (0.21–1.62)	0.305	0.22 (0.03–1.59)	0.133

^1^ Estimates obtained from multivariable Cox regression modeling including terms for including anatomic site, major gland subtype, age, sex, race, and receipt of adjuvant radiotherapy, ^2^ Disease-specific survival data were available for 1294 patients. HR: Hazard ratio; CI: Confidence interval.

**Table 5:** Association of major gland subsite and stage with overall and disease-specific survival in patients diagnosed with salivary gland ACC in the SEER Database (2004–2015).

		Stage I		Stage II		Stage III		Stage IV	
		HR ^1^ (95% CI)	*p*	HR ^1^ (95% CI)	*p*	HR ^1^ (95% CI)	*p*	HR ^1^ (95% CI)	*p*
Parotid[M14] [AAL15]	OS	referent		referent		referent		referent	
	DSS	referent		referent		referent		referent	
Submandibular	OS	0.77 (0.36–1.64)	0.498	1.00 (0.52–1.89)	0.992	1.43 (0.77–2.64)	0.256	1.61 (1.01–2.56)	0.044
	DSS	0.72 (0.21–2.55)	0.614	0.73 (0.26–2.06)	0.558	1.39 (0.61–3.17)	0.430	1.93 (1.13–3.30)	0.016
Sublingual	OS	N/A ^2^		0.97 (0.28–3.41)	0.966	0.64 (0.14–2.85)	0.559	0.68 (0.16–2.87)	0.595
	DSS	N/A ^2^		1.52 (0.36–6.38)	0.568	N/A^2^		0.44 (0.06–3.33)	0.428

^1^ Hazard ratio (HR) estimates were obtained from multivariable Cox regression modeling including terms for age, sex, and race. ^2^ Not estimable or not presented if fewer than 10 cases were present in stratum. OS, overall survival; DSS, Disease specific (salivary gland adenoid cystic carcino-ma)-specific survival.

**Table 6:** Impact of adjuvant radiotherapy on survival by gland type ^1^, tumor site, and stage, in patients diagnosed with salivary gland ACC in the SEER Database (2004–2015).

		Stage I		Stage II		Early-Stage		Stage III		Stage IV		Late-Stage	
		HR ^2^	95% CI	HR ^2^	95% CI	HR ^2^	95% CI	HR ^2^	95% CI	HR ^2^	95% CI	HR ^2^	95% CI
Minor	OS	0.93	0.40–2.18	1.76	0.64–4.85	1.39	0.77–2.53	N/A ^3^	.	0.90	0.47–1.73	1.03	0.55–1.92
	DSS	1.65	0.43–6.35	2.27	0.35–14.81	1.83	0.64–5.27	N/A ^3^	.	1.11	0.47–2.60	1.18	0.53–2.65
Parotid	OS	2.40	0.50–11.41	0.85	0.33–2.22	1.12	0.51–2.46	0.85	0.25–2.89	0.66	0.34–1.30	0.60	0.33–1.09
	DSS	N/A ^3^	.	1.69	0.30–9.42	2.47	0.53–11.46	N/A ^3^	.	0.55	0.23–1.34	0.66	0.28–1.55
Submandibular	OS	0.64	0.22–1.85	0.63	0.22–1.79	0.78	0.38–1.58	0.35	0.15–0.85	1.05	0.40–2.77	0.55	0.29–1.03
	DSS	0.42	0.61–2.83	0.55	0.10–2.94	0.63	0.19–2.08	0.54	0.13–2.17	N/A ^3^	.	0.64	0.26–1.57

^1^ The numbers of sublingual ACC cases were insufficient for TNM-stratified survival analysis. ^2^ Hazard ratio (HR) estimates were obtained from multivariable Cox regression modeling including terms for age, sex, and race. ^3^ Not estimable or not presented if fewer than 10 cases were present in stratum. OS, overall survival; DSS, disease specific (salivary gland adenoid cystic carcinoma)-specific survival.

## Data Availability

Publicly available datasets were analyzed in this study. Data can be found here: [https://seer.cancer.gov/].

## Appendix A

### Primary Site Algorithm in SEER Stat 8.3.5—m-ACC

(Site and Morphology.Primary Site—labeled) = ‘C00.0-External upper lip’,’C00.1-External lower lip’,’C00.2-External lip, NOS’,’C00.3-Mucosa of upper lip’,’C00.4-Mucosa of lower lip’,’C00.5-Mucosa of lip, NOS’,’C00.6-Commissure of lip’,’C00.8-Overlapping lesion of lip’,’C00.9-Lip, NOS’,’C01.9-Base of tongue, NOS’,’C02.0-Dorsal surface of tongue, NOS’,’C02.1-Border of tongue’,’C02.2-Ventral surface of tongue, NOS’,’C02.3-Anterior 2/3 of tongue, NOS’,’C02.4-Lingual tonsil’,’C02.8-Overlapping lesion of tongue’,’C02.9-Tongue, NOS’,’C03.0-Upper gum’,’C03.1-Lower gum’,’C03.9-Gum, NOS’,’C04.0-Anterior floor of mouth’,’C04.1-Lateral floor of mouth’,’C04.8-Overlapping lesion of floor of mouth’,’C04.9-Floor of mouth, NOS’,’C05.0-Hard palate’,’C05.1-Soft palate, NOS’,’C05.2-Uvula’,’C05.8-Overlapping lesion of palate’,’C05.9-Palate, NOS’,’C06.0-Cheek mucosa’,’C06.1-Vestibule of mouth’,’C06.2-Retromolar area’,’C06.8-Overlapping lesion of other & unspecified mouth’,’C06.9-Mouth, NOS’ AND (Site and Morphology.ICD-O-3 Hist/behav) = ‘8200/3: Adenoid cystic carcinoma’.

## Appendix B

### Primary Site Algorithm in SEER Stat 8.3.5—M-ACC

(Site and Morphology.Primary Site—labeled) = ‘C07.9-Parotid gland’,’C08.0-Submandibular gland’,’C08.1-Sublingual gland’ AND (Site and Morphology.ICD-O-3 Hist/behav) = ‘8200/3: Adenoid cystic carcinoma’.
